# Accelerating the Appropriate Adoption of Artificial Intelligence in Health Care: Protocol for a Multistepped Approach

**DOI:** 10.2196/30940

**Published:** 2021-10-06

**Authors:** David Wiljer, Mohammad Salhia, Elham Dolatabadi, Azra Dhalla, Caitlin Gillan, Dalia Al-Mouaswas, Ethan Jackson, Jacqueline Waldorf, Jane Mattson, Megan Clare, Nadim Lalani, Rebecca Charow, Sarmini Balakumar, Sarah Younus, Tharshini Jeyakumar, Wanda Peteanu, Walter Tavares

**Affiliations:** 1 University Health Network Toronto, ON Canada; 2 Institute of Health Policy, Management and Evaluation, Dalla Lana School of Public Health, University of Toronto Toronto, ON Canada; 3 Faculty of Medicine, University of Toronto Toronto, ON Canada; 4 Centre for Addictions and Mental Health, CAMH Education Toronto, ON Canada; 5 Michener Institute of Education at University Health Network Toronto, ON Canada; 6 Vector Institute Toronto, ON Canada; 7 Wilson Centre Toronto, ON Canada

**Keywords:** artificial intelligence, health care providers, education, learning, patient care, adoption, mHealth

## Abstract

**Background:**

Significant investments and advances in health care technologies and practices have created a need for digital and data-literate health care providers. Artificial intelligence (AI) algorithms transform the analysis, diagnosis, and treatment of medical conditions. Complex and massive data sets are informing significant health care decisions and clinical practices. The ability to read, manage, and interpret large data sets to provide data-driven care and to protect patient privacy are increasingly critical skills for today’s health care providers.

**Objective:**

The aim of this study is to accelerate the appropriate adoption of data-driven and AI-enhanced care by focusing on the mindsets, skillsets, and toolsets of point-of-care health providers and their leaders in the health system.

**Methods:**

To accelerate the adoption of AI and the need for organizational change at a national level, our multistepped approach includes creating awareness and capacity building, learning through innovation and adoption, developing appropriate and strategic partnerships, and building effective knowledge exchange initiatives. Education interventions designed to adapt knowledge to the local context and address any challenges to knowledge use include engagement activities to increase awareness, educational curricula for health care providers and leaders, and the development of a coaching and practice-based innovation hub. Framed by the Knowledge-to-Action framework, we are currently in the knowledge creation stage to inform the curricula for each deliverable. An environmental scan and scoping review were conducted to understand the current state of AI education programs as reported in the academic literature.

**Results:**

The environmental scan identified 24 AI-accredited programs specific to health providers, of which 11 were from the United States, 6 from Canada, 4 from the United Kingdom, and 3 from Asian countries. The most common curriculum topics across the environmental scan and scoping review included AI fundamentals, applications of AI, applied machine learning in health care, ethics, data science, and challenges to and opportunities for using AI.

**Conclusions:**

Technologies are advancing more rapidly than organizations, and professionals can adopt and adapt to them. To help shape AI practices, health care providers must have the skills and abilities to initiate change and shape the future of their discipline and practices for advancing high-quality care within the digital ecosystem.

**International Registered Report Identifier (IRRID):**

PRR1-10.2196/30940

## Introduction

### Background

The convergence of information technology and medicine in our dynamic digital era has produced novel applicable solutions for clinical practice [1]. Health care is at an important turning point for the efficient and safe use of artificial intelligence (AI) technologies to transform the quality of care delivered [2]. AI is an expansive term for various systems or technologies that originate from the branch of computer science. Such technologies are designed to emulate and augment human intelligence under headings, such as visual perception, reasoning, learning, speech recognition, and the ability to perform human tasks [3-5]. Machine learning is a subset of AI that depends on algorithms to enable computers to learn patterns and rules by using previous examples [4,6]. AI can be leveraged to help deliver precision medicine, optimize patient and clinical outcomes, reduce costs, and enhance the efficiency and accessibility of the health care system [2]. Despite great technological advances, little is known about how to put this new knowledge and these new tools into practice.

There is emerging evidence that AI has the power to transform care delivery in many domains [7], and recent advancements and breakthroughs in machine learning techniques involving deep neural networks trained on big data have increased the adoption of AI in health care [8]. The extent to which AI technologies could alter the landscape of prevention, diagnosis, medical care, and predictive health services is significant, as well as improve the delivery and effectiveness of health care [9]. AI can play a significant role in this process, given the potential to recognize subtle disease-specific patterns from various sources that humans would never recognize [10]. For instance, AI is currently being used to reduce false-positive results in screening for breast cancer, to revolutionize clinicians’ workflow and robotic surgery, and to predict mortality rates of patients [10]. A study by Google Health demonstrated AI’s ability to perform better than human experts in breast cancer prediction [11]. Consequently, implementation of this AI system may influence recall rates, the number of unnecessary biopsies, and earlier detection of cancer due to the specificity and sensitivity improvements in identifying invasive cancers. The era of AI envisages new roles of care providers; thus, health professional education and clinical practice will experience profound change. [12].

The implementation of AI is perceived favourably, even enthusiastically by patients due to the possibility of greater engagement and personalized treatment. However, it is often encountered with trepidation from health care providers who are not prepared for such an evolution of practice [5,13]. According to Briganti and Le Moine, this trepidation can be attributed to four widely discussed reasons [13]. First, owing to the lack of education in AI, health care providers may feel unprepared to adopt AI in their clinical settings [13]. Second, there is an increased administrative burden associated with a shift in the emergence of technologies being used to support healthcare processes such as electronic health records (EHRs). This can contribute to clinician burnout [13]. Third, many clinicians fear that these new tools will replace their roles, even though emerging academic literature highlights that AI will be used in conjunction to support clinicians in making decisions [13]. Finally, it is imperative to note that the current legal structure does not specify the terms of liability in the event of adoption or rejection of algorithm recommendation, which can leave the care provider exposed to potential legal outcomes [13].

There are numerous barriers in understanding the potential of AI in health care ecosystems [2]. Current challenges are centered on the application of AI to populations not depicted in the training and testing of data sets, use of biased data for the development of AI models, neglect toward the potential inadvertent consequences of care, and lack of data regarding the efficacy and effect on patient outcomes and the health care system [2]. The *black box* phenomenon encumbers the adoption of AI among the medical community because they ultimately have to make the final clinical decision without understanding of how the variables within the AI algorithm forms the prediction, in which their decision is based on [10]. Thus, it is difficult for clinicians to trust the algorithm, and in turn, become accustom to working with AI [10]. Health care providers should leverage technology [14] and advocate for the ethical use of patient data and AI to maintain trust with their patients and enhanced, equitable care [15]. As the health care community prepares for this shift with a sustained commitment to upholding high standards of care, it is crucial to refocus medical education on developing medical innovators [16].

The emergence of AI offers unprecedented opportunities for accelerating scientific advances in health care; however, current educational structures are not sufficient to prepare care providers to leverage those opportunities. Health care providers often face deficits in the knowledge and skills needed to provide optimal care for all patients [17]. Lifelong learning has been defined in the literature as the sustained motivation to pursue self-initiated learning activities and having the required information-seeking skills, and the ability to identify one’s own learning needs [18]. The literature on implementing health information systems (HISs) indicated that adequate education efforts had a favorable impact on clinicians’ views on HIS adoption and their ongoing use of the system [19,20]. A study by Bredfeldt et al [20] highlighted that education was associated with increased use of key EHR features for medication list management, an addition to the meaningful use criteria. Similarly, Kraus et al [19] reported that physician adoption with HIS reached 40% in the first month and stabilized at 75% within a year. The authors asserted that ensuring success requires a systematic approach to change management, including training, workflow redesign, and support during the transition [19]. Embedding AI concepts as part of knowledge translation could be a fundamental step in equipping care providers to engage in competent and safe practices [4]. Chaumunyonga [4] asserted the importance of equipping radiation therapy professionals with the knowledge and skills to participate in discussions about the use of AI and integrate AI in their practice where quality and safety standards are maintained. Given the importance and potential impact of AI, knowledge translation products should include content that will allow care providers to translate knowledge of emerging technologies into their own practice [21]. Sit and colleagues reported that it is vital to seize this opportunity to prepare a health care workforce with adequate knowledge and skills to effectively use new digital tools, including AI technologies [22]. The curricula should be reformed to meet the needs of the changing health care system [23].

### Objective

The objective of this project is to accelerate the appropriate adoption of data-driven and AI-enhanced care by focusing on the mindsets, skillsets, and toolsets of point-of-care health providers and their leaders in the health system.

Specifically, we aim to (1) develop and evaluate knowledge translation interventions to harness data and AI to enhance and optimize health care delivery, (2) examine the contextual factors that influence the success of the program and the adoption of AI initiatives in health care, and (3) explore the barriers to and facilitators of the implementation of an AI education program.

## Methods

### Overall Study Design

**Figure 1:**
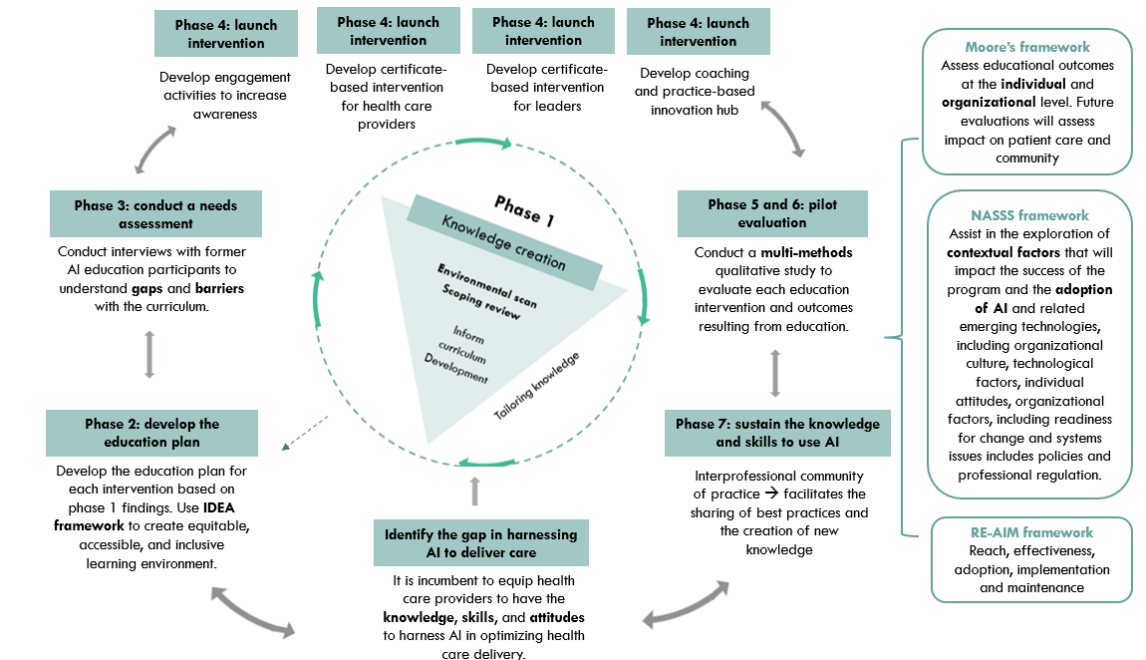
Overall study design adapted from the study by Graham et al [26] with permission. AI: artificial intelligence; IDEA: inclusion, diversity, equity, and accessibility framework; NASSS: Nonadoption, Abandonment, and Challenges to Scale-up, Spread, and Sustainability.

In taking an integrative knowledge translation approach, this project will be framed by the Knowledge-to-Action (KTA) framework [24,25] for transformational change in the health care system through the integration and developmental evaluation of evidence-based AI education interventions (Figure 1).

The knowledge funnel represents the refinement of the knowledge base to inform the knowledge product development, whereby first the knowledge is created, then synthesized and interpreted [26]. Knowledge synthesis involves the identification, appraisal, and aggregation of studies or information relevant to the research questions [26]. The action part of the process is represented by the outer circle, which consists of activities leading to the effective implementation of knowledge [26].

### Evaluation Approach

The research will examine the knowledge uptake and barriers to AI adoption in clinical settings using quantitative and qualitative methodologies. Table 1 illustrates the frameworks used in each phase as part of the evaluation process.

**Figure 2:**
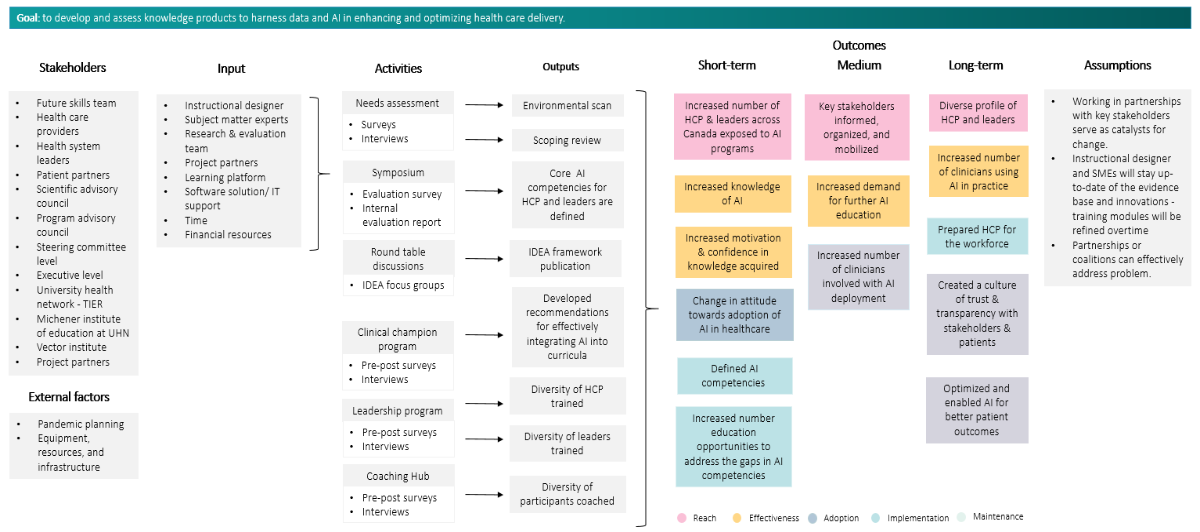
Logic model. AI: artificial intelligence; HCP: health care provider; IDEA: inclusion, diversity, equity, and accessibility framework; IT: information technology; SME: subject matter expert; TIER: The Institution for Education Research; UHN: University Health Network.

A logic model was developed to describe the intervention design and guide the evaluation plan (Figure 2). The activities and outputs mentioned in the logic model were expanded in each phase of the KTA framework.

**Table 1:** Cocreating the knowledge translation method.

Knowledge translation phase	Aim 1 (Moore framework)	Aim 2 (NASSS^a^ framework)	Aim 3 (RE-AIM^b^ framework)
Phase 1: identify the problem	✓^c^		
Phase 2: adapt to the knowledge			✓
Phase 3: assess barriers and facilitators			✓
Phase 4: launch the intervention	✓	✓	
Phases 5 and 6: pilot evaluation	✓	✓	✓
Phase 7: sustain ongoing knowledge use		✓	✓

^a^NASSS: Nonadoption, Abandonment, and Challenges to Scale-up, Spread, and Sustainability.

^b^RE-AIM: Reach, Effectiveness, Adoption, Implementation, and Maintenance.

^c^Phase to be evaluated.

### Phase 1: Identify the Problem and Create Knowledge

In this first phase, our aim is to identify the problem and the knowledge required to address this gap. The health care ecosystem is witnessing a surge in AI-powered digital health technologies that can potentially augment the delivery of care and affect population health [27]. Although the advent of data technologies has led to numerous opportunities to transform the evolving arenas of care, organizations are much slower in embracing technological changes [28,29]. Brinker summarized this concept of technologies changing exponentially as organizations change logarithmically, which is known as the Martec law [28,29]. This discrepancy can lead to a widening gap in how organizations and care providers can harness the potential of AI technologies to enhance practice and care delivery [28,29]. To address this gap, Brinker suggests that health care organizations must address how those technologies will be integrated into the operations and culture of an organization [29]. This point was reinforced in the review by Topol [7], who reported that an effective culture of learning is needed to enable the workforce to reframe their knowledge and become digitally competent and confident within an increasingly technology-driven environment. The interventions we develop will not only increase knowledge, but also provide a multidisciplinary audience with the required tools to lead conversations around shifts in practice, policy, procedures, and problem-solving approaches that optimize this new digital presence.

An evidence-based education and coaching program will be developed for health care professionals to build the knowledge, skills, and capabilities to translate the potential power of AI applications into effective practice. First, we will plan, develop, and scale education interventions designed to create awareness and change mindsets regarding the appropriate use and applications of AI. The shift in mindset will explore equity, social responsibility, advocacy, data governance, and transparency for AI use. Second, we will build advanced AI literacy skills for care providers and leaders by providing access to high-quality education interventions that build the capability for augmented intelligence through AI. Finally, a coaching and practice-based innovation hub will be implemented to support practice change and build self-efficacy to use AI in practice. These educational interventions will be built on what is learned through an environmental scan, scoping review, and a needs assessment, all of which will inform curricular content, delivery and implementation approaches, and programming outcomes.

### Phase 2: Adapt Knowledge

In this second phase, our aim will be to ensure that we adapt the knowledge to the local context and consider strategies that help to design and develop equitable, diverse, and inclusive knowledge translation products. The increasing adoption of AI tools in the past decade presents an opportunity for a paradigm shift in health care toward economical, integrated, and equitable care delivery [27]. This shift can be seen through the lens of the National Academy of Medicine Quintuple Aim, focusing on various domains of health care improvement and equity. There are opportunities to build and focus on AI to reduce cost and improve population health, care team well-being, patient experience, and equity and inclusion [27]. The affordability of remote monitoring devices increased web-based care, and real time feedback tools position AI as an essential factor in achieving the quintuple aim of health care [27]. The authors further emphasized the need for significant retooling of the health workforce and the required shifts in entry to practice [27]. The National Academy of Medicine places a particular focus on mitigating the risk that AI might lead to a less equitable delivery of services, and we will follow its recommendations on education for AI-enabled equity and diversity [27]. Specifically, the framework will be used to guide the implementation and evaluation of AI through an equity and diversity lens, evaluating data biases, trust and transparency factors, and the inclusion of the social determinants of health. With a focus on diversity and equity as key principles in the study design, the evidence generated through this initiative will be applicable to many settings and will inform evidence-based approaches to the adoption of AI in other industries critical to the promotion of health and better outcomes.

To ensure meaningful and impactful learning experiences, our knowledge development processes will be guided by an equity and inclusion lens that not only promotes diversity but also eliminates barriers to learning. An inclusion, diversity, equity, and accessibility (IDEA) framework that demonstrates the intersection between education development and IDEA principles will be the cornerstone of this knowledge to practice initiatives. Developing education using this framework will ensure that instructional designers, subject matter experts, and faculty members reflect on and question their biases and assumptions throughout the education development, delivery, and evaluation cycle. Explicit reflections are critical for eliminating biases in dominant educational cultures that marginalize certain populations [30]. An equity lens in design is also foundational to building programs that are safe and accessible to diverse learner populations [31]. Within the context of the COVID-19 pandemic, our programs will be delivered through a web-based format. Unlike in-person learning, we recognize that web-based learning tends to be built on Eurocentric and ableist paradigms [30] and that web-based delivery may impede the equitable and accessible distribution of new knowledge. Therefore, leveraging the IDEA framework will afford the intentional integration of accessible and equitable practices into web-based delivery. To this end, the use of universal design learning for accessible and flexible learning is intended to build a web-based environment that is inclusive of all learners, harnesses the strengths of learner diversity, includes principles of culture and intersectionality, and creates a sense of belonging for all participants [30,32].

### Phase 3: Assess Barriers and Facilitators

#### Overview

In the third phase, we aim to identify potential barriers and facilitators related to the adoption of AI in health care. As part of the needs assessment, a qualitative study will be conducted with former AI education participants and patient partners to understand the gaps and barriers with the current AI curriculum for health care providers. The findings will enable us to identify determinants of the evidence-practice gap, specific factors that influence the adoption and implementation of AI in clinical settings, and the specific targets to be addressed by our education interventions. This will generate a thorough understanding of health care providers’ and patients’ perceptions in enhancing and optimizing health care delivery through the use of AI. A survey will also be disseminated to health care providers across Canada to better understand learners’ needs when developing the curriculum.

#### Needs Assessment: Study Population and Sampling

Key informants will comprise health care providers (any practice setting), leaders, and scientists who have taken part in an AI education program, as well as patient partners. Health system leaders are those enabling and empowering other professionals to integrate AI into the health care setting. We anticipated needing 30 to 40 interviews with key informants given the variety of perspectives, different health professions, and geographic location [33].

#### Needs Assessment: Recruitment Procedure

##### Qualitative Interviews: Existing AI Programs Offered Through Michener Institute of Education at University Health Network and Vector Institute

The Michener Institute of Education at University Health Network (UHN) and the Vector Institute offer several AI programs for equipping learners with AI skills and competencies sought by the industry. This includes the AI Certificate program and the Special Topics course at the Michener Institute of Education at UHN [34], as well as the AI programs accredited by the Vector Institute, such as the University of Toronto’s Master of Health Informatics program. A purposive sampling approach will be used to ensure that there is diversity in the types of health professionals and executives, age, years in practice, locations of practice (urban vs rural), and types of health organization (academic, community, and independent practice). We will reach out to the Michener Institute of Education at UHN and the Vector Institute leads, part of this project team, for a list of participants who have taken part in an AI program that was targeted for health care providers and leaders at their institutions.

##### Qualitative Interviews: Environmental Scan and Scoping Review

AI programs will be identified through an environmental scan and scoping review, the purpose of which is to understand the current landscape of AI education programs and gain important insights into successful education development. The program leads for courses that are publicly listed on their websites will be contacted and asked to send email invitations to their educators and learners on behalf of the project team. Three to five participants will be interviewed at each organization.

##### Qualitative Interviews: Project Partners and Snowball Sampling

In addition, participants will be recruited via partner organizations for this project. This will include email invitations sent on behalf of the research team by the education committee members of those organizations. Those who have consented to be part of the study will be asked to inform colleagues within their networks and share the research analyst’s contact information with those interested in participating.

##### Qualitative Interviews: Patient Partners

Patient partners will also be purposively sampled through partner organizations. Discussions with patient partners will help us to understand where there are gaps in AI education for care providers and where they would like them to be knowledgeable about using AI. It is imperative to consider patient partner input when creating content relating to patient interactions and considerations.

##### Survey: Partner Organizations

The survey will be disseminated through our partner organizations. We will also reach out to our community of interest that we are building for this project, such as through the events hosted and the sign-up list. When registering for the event, they have the option to receive news about upcoming initiatives, tools, and learning opportunities. There is also a sign-up list on the Michener Institute of Education at UHN website for individuals to sign-up to receive electronic communications about the project.

#### Needs Assessment: Data Collection

##### Qualitative Interviews

We will conduct semistructured interviews with key informants; this methodology will allow for further exploration of any issues participants may reveal as significant. An interview guide will be used to review participant experiences and suggestions for developing AI curricula, which will include questions on motivations for AI education program registration, the relevance of subject material to participants, and barriers to engagement with further AI-related education initiatives. This guide will be revised iteratively with each interview, as necessary. Each interview will be conducted and, recorded through Microsoft 365 Teams app and transcribed using NVivo 12 (QSR International). Interview length may vary based on the participant’s level of comfort in sharing their experiences.

##### Survey

A survey will be disseminated to health care professionals across Canada to better understand their perceptions and adoption of AI in clinical practice.

#### Needs Assessment: Data Analysis

##### Qualitative Interviews

Transcripts will be analyzed thematically using an iterative, inductive, and constant comparative process. The flexibility of the thematic analysis allows the researcher to report the experiences, beliefs, and reality of participants in rich detail [35]. Most importantly, it establishes a more systematic and explicit form of examining data without losing the rigor of the analysis [35]. Two study reviewers will analyze the transcripts independently from the first three interviews to identify codes and shape further data collection. The team will collaboratively develop an initial coding structure. Data will be inductively analyzed following the systematic process outlined by Braun and Clarke [35]. New data will be constantly compared with existing data, resulting in an iterative refinement of the coding structure. Any discrepancies in coding will be discussed with the team until a consensus is reached. QSR NVivo 12, a qualitative data analysis software program, will be used to examine responses to emerging and recurring themes. Data collection and analysis will continue until theoretical saturation is reached. To ensure transparency and rigor, we will collect field notes, and an audit trail of each team member’s independent coding, team meeting notes, and different versions of the coding structure will be maintained. In addition, the quality of the thematic analysis will be assessed using Braun and Clarke’s [36] twenty-question evaluation tool.

##### Survey

Descriptive statistics will be used to analyze the close-ended quantitative survey data. Software program GNU Affero General Public License R Studio and IBM SPSS will be used to report the descriptive statistics. Content analysis will be used to analyze open-ended qualitative survey data in two distinct phases: deductive and inductive.

### Phase 4: Launch Intervention

#### Overview

In phase 4, our aim will be to develop knowledge translation products that promote awareness and the use of knowledge to harness data and AI to enhance care delivery. In a recent Harvard Business Review article, Fountaine et al [37] argued that culture, rather than technology, is a major challenge in adopting AI. The authors provided guidelines on establishing internal structures and proposed a flexible hub and spoke model, which will help organizations scale. This model focuses on expertise that is both centralized to ensure consistency in strategies, procedures, and partnerships and decentralized to ensure that the work is ingrained in essential business activities and performance improvement [28,37]. This fluid model is vague in terms of responsibility. Depending on an organization’s structures, capabilities, strategies, and unique characteristics, work can be transitioned between centralized and decentralized responsibilities [6,28]. This study further highlights the need to educate everyone. The type of education required is based on an individual’s role; this can be a formidable task within the context of the exponential growth of digital technologies and the growing divide between the emergence of technologies and organizational readiness [28,37]. Most health care providers have not been educated on the effective, appropriate, safe, and compassionate use of AI, yet they must safely adopt these tools and shift their scope of practice. The process of implementing AI-enabled technologies in health care organizations must be prudently considered as care providers have the obligation to do no harm [28]. Health care providers should have the necessary knowledge to shape the future of AI-enabled care.

**Figure 3:**
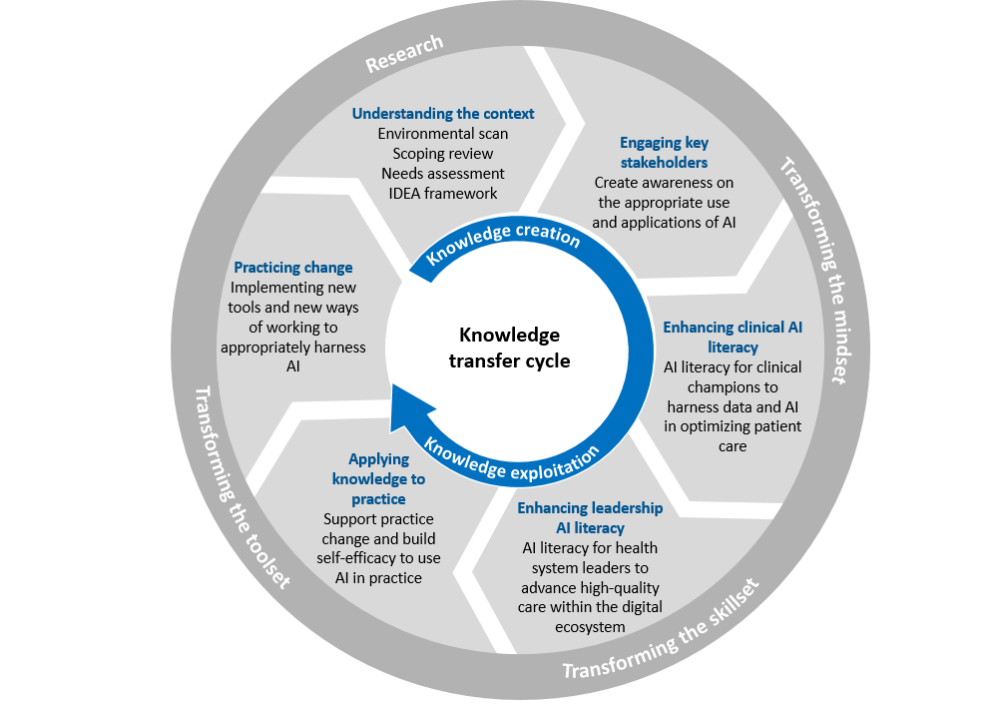
Knowledge translation interventions. AI: artificial intelligence; IDEA: inclusion, diversity, equity, and accessibility framework.

Using the data obtained from phases 1 to 3, we will develop four knowledge translation interventions that will accelerate the rate of organizational change and ensure that AI enhances and optimizes health care delivery (Figure 3). The interventions include engagement activities to increase AI awareness (intervention A), a certificate-based intervention to educate health care providers about AI (intervention B), a certificate-based intervention to educate health system leaders about AI (intervention C), and a coaching and practice-based innovation hub (intervention D). The interventions will help increase health care providers’ and leaders’ knowledge, confidence, and skills in integrating AI as part of their practice.

The program design and delivery in the pilot phase will be based on empirical evidence and expert consensus of curricula content, specifically, what is feasible in our specific context. The proposed program will be designed to transform the mindsets, skillsets, and toolsets of health care providers, thereby accelerating the appropriate adoption of AI. The curricula will be developed with an instructional designer through an iterative process that follows the successive approximation model [38]. Subject matter experts and other stakeholders will be engaged during the design and development process to provide feedback and continuously improve curricula. Before the release of the final version of the educational interventions, usability testing will be conducted to ensure that end-user perceptions, requirements, and information needs are met. Usability testing is crucial for identifying errors, participant decision-making, and reasoning skills, as they perform specific tasks. Usability testing will be guided by Nielsen’s 10 Usability Heuristic Principles and Severity Scale. The heuristic analysis will serve as an evaluation method to examine the user interface and to identify issues so that they can be resolved, thereby improving user satisfaction and experience. The findings will be used to further tailor and iteratively refine the program (see Table 2 for the prospective design of the education interventions)*.*

**Table 2:** Prospective design of education interventions.

Education intervention	Target audience (sample size)	Prospective format
A: Engagement activities to increase awareness	5000 participants	Synchronous and asynchronous
B: Certificate-based intervention to educate health care providers about AI^a^	25-50 health care providers	Synchronous and asynchronous 4-6 modules Closed registration
C: Certificate-based intervention to educate health system leaders about AI	25-50 health system leaders	Synchronous and asynchronous 4-6 modules Closed registration
D: Coaching and practice-based innovation hub	20 teams within AI projects	Community of practice Goal setting and reflection-based activity Closed registration

^a^AI: artificial intelligence.

#### Education Interventions A-C

The interventions will be designed and iteratively adapted based on learners’ needs identified through knowledge creation activities. These interventions are anticipated to incorporate both synchronous and asynchronous elements so that learners can prepare for, and engage in, interactively. Instructional strategies include reflection exercises, participant-led discussions, and sharing of course materials. Prospective curriculum topics include AI fundamentals, AI in health care, ethics, data science, and the challenges to and opportunities for using AI.

#### Education Intervention D

The coaching and practice-based hub will support health care providers in implementing high-impact projects, thus building momentum for AI-enabled care. The hub leverages the structure of our in-house personalized learning program [39], which is a fully personalized and engaging observership-based program intended to meet learner goals [39]. The personalized learning program structure ensures that no two programs are the same, as each project or learner presents unique needs. By leveraging this structure, we will also use various resources and expertise throughout the country to align with the learners’ professional development goals. Coaching and mentoring time and education opportunities, such as workshops will be scheduled within the education plan as applicable. This opportunity will provide learners with an immersive experience, in an environment with expertise in both AI adoption and implementation, and teams that will support the learners by linking their learning to their own institution and projects. Learners will be matched with an expert coach. With this experience, we hope to give learners the confidence to take great ideas and adapt them to fit into their environments. This experience will also be an opportunity to network with AI leaders across the country.

There is an imperative need to mobilize knowledge across professions and organizations to address the gap between technological change and organizational readiness. Web-based learning can expand the reach of our project’s coaching and practice-based intervention, which may enable a more diverse cross-section of projects that can be coached. This expanded reach will enable an interprofessional community of practice (CoP), developed initially by Wenger et al [40]. CoP provides an ideal setting to identify knowledge and skill gaps to pursue learning with colleagues and experts to fill these gaps. Li et al [41] highlighted several key characteristics to guide the development of a community practice, which include formal and informal interaction between the learners and the experts, importance on learning and sharing knowledge, and nurturing a sense of acceptance among members of the community. The authors further asserted that a learning community provides a safe environment for individuals to engage in learning through collaborative discussions with other members and experts in the field [41]. This research will contribute to our understanding of the role and impact of CoPs in translating AI knowledge into practice.

### Phases 5 and 6: Pilot Evaluation

#### Overview

In phases 5 and 6, a pilot evaluation will be conducted to evaluate each educational intervention and to determine if the desired outcomes are achieved. A multimethod evaluation approach will be guided by the Reach, Effectiveness, Adoption, Implementation, and Maintenance (RE-AIM) framework [42]. The RE-AIM framework will enable us to assess whether the interventions are effective in enabling care providers to harness AI in optimizing health care delivery, and the curricula are reflective of best practices. The survey questions will be guided by the RE-AIM framework to measure the reach, effectiveness, adoption, implementation, and maintenance of educational interventions [42]. Interventions will be iteratively updated and refined using a developmental evaluation strategy. Interventions A to D will be observational pre- and posttest methods, including mixed methods design consisting of surveys and interviews to understand and assess the impact of the intervention from a quality improvement perspective. In addition, intervention D will consist of goal setting and reflection exercises (Table 3 lists the research methods)*.*

**Table 3:** Multimethods for evaluating education interventions.

Aim and data collection format		Education intervention
**Understand why certain curriculum topics are important and the level of receptivity toward and engagement in implementation of AI^a^ in their practice setting**		
	Surveys Interviews or focus groups	A: engagement activities to increase AI awareness
**Explore their perceptions of the program, whether it influenced them to seek out further learning, identify curriculum topics of interest, topics to prioritize, and their professional background**		
	Pre- and postsurvey Interviews or focus groups	B: health care providers
	Pre- and postsurvey Interviews or focus groups	C: health system leaders
	Pre- and postsurvey Reflections Interviews or focus groups	D: coaching and practice-based innovation hub

^a^AI: artificial intelligence.

#### Study Population and Sampling

For education interventions A to D, participants will include any individuals 18 years or older who have registered for the intervention and have shared their names, full mailing addresses, and email addresses. Engagement activities are intended to create awareness and increase the ability of health care providers to engage in AI-based initiatives. In addition, there will be two certificate-based interventions developed specifically for health care providers and leaders. Registration is open to health care professionals with a particular focus on those working in the health system. Health care professionals are defined as staff who provide health care services to patients and include clinical, administrative, and other nonclinical staff. Health system leaders are considered champions who will be actively involved in essential conversations at all levels of the organization and help put AI into practice. For the coaching and practice-based intervention, participants would include health care providers and leaders working in the National Health Care System and those who have an interest or identify the need to advance their data literacy and AI knowledge. Eligibility criteria also include the ability to have advanced proficiency in reading, writing, and speaking English, because all components of these activities are available only in English. The response rate is not anticipated to be affected by this English proficiency criterion. Engagement materials with participants will be offered in English and French; however, the evaluation components will only be in English.

#### Recruitment Procedure

 Prospective program participants (for interventions B-D), including health care providers and health system leaders, will be recruited using various channels, such as national specialty societies across health professions and leadership associations [43-44]. Participants can also self-identify through earlier project activities, such as needs assessment. For the coaching and practice-based innovation hub (intervention D), participants in the coaching will also be recruited from AI certificate interventions developed by us, and via the Michener Institute of Education’s and the Vector Institute’s social media platforms. Potential candidates will be selected through an interview process and matched with a coach on the basis of learning objectives and area or areas of expertise. Coaches for intervention D will be recruited from a pool of experts and leaders in AI faculty from the certificate programs we developed as well as through provincial AI incubators and think tanks. Coaches are required to provide their expert profiles to facilitate the matching process.

#### Data Collection

##### Pre- and Postsurvey

The quantitative phase consisted of a pre- and postsurvey for each education intervention evaluation. The presurvey consists of three sections: (1) demographics and practice context, (2) engagement in AI-related education, and (3) motivation level for learning. The motivation level for learning will be measured using the Jefferson Scale of Physician Lifelong Learning (JeffSPLL), which consists of 14 items, and generates scores ranging from 14 to 56. Higher scores denote greater affinity for lifelong learning [45]. The JeffSPLL consists of three factors: learning beliefs and motivation, attention to learning opportunities, and information-seeking technical skills. The reliability of the JeffSPLL scale is 0.77 to 0.86 in the previous studies [45].

The post survey consists of two sections: (1) experiences with the program and (2) usability of the educational activities offered on the web. The surveys were structured based on the Moore framework [46]. Questions are both quantitative and qualitative (ie, open responses) in nature. The majority of the survey is quantitative, requiring participants to select from a radio list of options, or a 5-point Likert scale ranging from *strongly disagree* to *strongly agree*. For instance, in section 1 (thoughts about the program), participants will answer the following questions using a 5-point Likert scale for each program: (1) “My knowledge and awareness of this topic increased;” (2) “The topics covered in this program are relevant to me.” Examples of qualitative or open-ended questions for section 1 include, “which topics were missing or you wish were covered in more depth and why?” In section 3, the System Usability Scale (SUS) is used to measure the usability of educational activities offered on the web [47]. The SUS is a 10-item questionnaire with five response options and generates a score ranging from 0 to 100. The average SUS score was 68; thus, higher scores indicated better usability.

##### Qualitative Interviews or Focus Groups and Reflection Exercises

The qualitative phase will consist of two data collection methods: semistructured interviews or focus groups for each education intervention evaluation. Semistructured interviews and web-based focus groups were chosen as the optimal methods because they provide the opportunity to capture richer descriptive data around participants’ behaviors, motivations, and experiences [48]. In addition, focus groups are an opportunity to allow participants to understand and hear differences of opinion and build on each other’s statements [49].

For interviews and focus groups, maximum variation and purposive sampling [50] will be used to explore the phenomena of interest across a range of demographically varied participants. Approximately, 15-20 participants from within the education program cohort will be selected to participate in a semistructured interview or focus group [33]. Questions will explore phase 1 findings to (1) understand the knowledge gain and applications of knowledge structured by the Moore framework [46]; (2) elicit feedback on the program curriculum, delivery, and format; and (3) understand contextual factors structured by the Nonadoption, Abandonment, and Challenges to Scale-up, Spread, and Sustainability framework [51]. The Moore framework will provide a structure to explore education outcomes at the individual, organizational, community, and system levels, with a particular focus on assessing the impact on patient care [46]. From a technological perspective, the Nonadoption, Abandonment, and Challenges to Scale-up, Spread, and Sustainability framework [51] will allow us to explore contextual factors that will affect the success of the program and the adoption of AI and related emerging technologies, including organizational culture, technological factors, individual attitudes, and organizational factors, including readiness for change and systems issues. Interview data will be deductively analyzed using the constructs from these frameworks as predefined codes. The interview guide will be iteratively enhanced during each interview conducted. Each interview will be audiotaped, and professionally transcribed.

Furthermore, for coaching and practice-based intervention, a goal assessment document will be used to set expectations and learning objectives. Reflection experience documents will be submitted by mentees at the 3- and 6-month marks. These documents will assist mentees in reflecting on their experiences and identifying areas of personal, professional, and academic growth***.*** The reflective approach is guided by the Kolb experiential learning theory. Kolb asserted that learning is a process in which knowledge is generated through experiences [52]. Specifically, immediate personal experience enables one to observe and reflect, which can be assimilated into abstract concepts, guiding learners to create new experiences [52]. Thus, the coaching and practice-based innovation hub built on the four stages of experiential learning theory would elicit evidence for changes in cognitive processes, learning, and behavior.

#### Data Analysis

##### Pre- and Postsurvey

Descriptive and content analyses will be used to determine: (1) who participated in this program; (2) if participants increased their knowledge of the scope of AI, automation, and machine learning in the Canadian context; (3) if participants received the information they were hoping for, and if not, what was missing; (4) of the information received, what was critical or most relevant; (5) if participating in this program prompted participants to seek further learning; and (6) if the participants planned to seek or participate in further learning. Descriptive statistics will be used to analyze the closed-ended survey questions, and an iterative, inductive method of constant comparative analysis will be used to analyze the open-ended responses [53]. Content analysis enables a systematic and objective means of describing the characteristics of a phenomenon [54,55]. Interview and focus group data will be analyzed using an open coding process consisting of meaningful words and phrases.

##### Qualitative Interviews or Focus Groups and Reflection Exercises

Audio recordings of interviews and focus groups will be transcribed, and open-ended responses to goal assessment and reflection experience documents will be collected. Data will be first analyzed deductively using the Moore framework [46]; predefined codes generated on the basis of the different outcomes of the Moore framework will then be inductively analyzed, followed by a systematic process outlined by Braun and Clarke [35]. Two study reviewers will independently analyze the transcripts from the first three interviews or focus groups to identify codes, and the team will collaboratively develop an initial coding structure. The coding structure will be iteratively refined by constantly comparing new data with existing data. Data collection and analysis will continue until theoretical saturation is reached. Any discrepancies in coding will be discussed with the team until a consensus is reached. Triangulation will be used to establish the themes. QSR NVivo 12 will be used to examine the responses to emerging and recurring themes.

### Phase 7: Sustain Ongoing Knowledge Use

Strategies to sustain ongoing knowledge use were considered in this project, including ensuring participation from important stakeholders and an evaluation to gain useful insights on emerging learning needs and support the optimization of educational interventions. As part of the coaching and practice-based innovation hub, an interprofessional CoP will have been established to share experiences and allow for the creation of new knowledge to advance the field of AI in health care. Hence, it is imperative to consider strategies on how the CoP will be sustained with former participants and engage prospective learners to build a dynamic web-based learning community. The findings will be used to inform appropriate dissemination strategies and the sustainability of the program.

### Governance

The governance model has an overarching goal of establishing and sustaining the alignment of strategic priorities to enhance transparency and communication pathways, and ultimately support the achievement of the deliverables set out in the Future Skills Grant project. The governance structure will follow a four-tiered model, in which day-to-day operations and activities related to individual projects will be discussed at the task force for decision-making and identification of issues requiring escalation. The task force will seek advice from the Scientific Advisory Council and the Program Development Advisory Council. Demand, ongoing initiatives, and escalations will be shared at the steering committee level for informational purposes, resolution, decision-making, and analysis of cross-project impact. Key items will be presented at the executive level, primarily to seek guidance in decision-making and to provide any ongoing updates. To put the needs of patients first in creating a healthier world using AI, we will involve patient partners in the design and development of our education programs. They will be engaged in defining the success of the programs and ensuring that they are meaningful to the target population.

## Results

This study is currently under review by the institutional review board of the UHN. Due to the large, complex nature of this initiative, the research ethics board requested that the submission be completed in phases. Informed, implied consent will be obtained based on the completion of the survey, and informed written consent will be obtained before proceeding with the semistructured interviews or focus groups. We will conduct preliminary data collection for educational interventions in 2021. These results are expected to be published in 2022.

## Discussion

### Short-term and Medium-term Outcomes

This proposed study will be designed to transform the mindsets, skillsets, and toolsets of health care providers across Canada, accelerating the appropriate adoption of AI. Preliminary results indicate that there is a need for national education standards, competency-based frameworks, and evaluation approaches. Participants will have increased knowledge of AI to have necessary and essential conversations at all levels of the organization. In addition, a framework on the capabilities and competencies of future AI workforce will be developed based on the findings from the needs assessment, including the environmental scan and scoping review. This study will develop knowledge translation interventions for the adoption and implementation of AI tools in clinical practice.

### Long-term Outcomes

In the long term, health care providers will have developed the required competencies and capabilities to adapt their practices in an AI-enabled environment. This project will create a culture of trust and transparency with stakeholders by establishing awareness and building the capacity and capability to have meaningful conversations about AI and its applicability. The interventions we developed assist organizations in educating health care providers and leaders to have access to knowledge products for the adoption and implementation of AI tools. The education program is designed to support organizations to more rapidly adopt AI technologies with confidence, knowing that health care providers have the appropriate knowledge and skills.

### Dissemination

Research activities are designed to engage key stakeholders, solicit feedback about the project, and disseminate findings. As part of the integrated knowledge translation and community-building efforts, the project team will be posting on social media and creating engagement activities such as AI blog posts, infographics, and video vignettes to establish awareness and engage potential participants in upcoming education programs. Stakeholders will be engaged and briefed at every stage of the KTA framework. The feedback will be used to further refine the programs to meet the learners’ needs. Dissemination is a part of a dynamic and iterative process that entails building relationships among key stakeholders. The final results will be disseminated via nontechnical briefs, round table discussions, symposia, conference presentations, and publications.

## Abbreviations

AI: artificial intelligence
CoP: community of practice
EHR: electronic health record
HIS: health information system
IDEA: inclusion, diversity, equity, and accessibility
JeffSPLL: Jefferson Scale of Physician Lifelong Learning
KTA: Knowledge-to-Action
RE-AIM: Reach, Effectiveness, Adoption, Implementation, and Maintenance
SUS: System Usability Scale
UHN: University Health Network
